# Supplementary figures and images for: Worldwide Patterns of Ancestry, Divergence, and Admixture in Domesticated Cattle
Source: PLoS Genet. 2014 Mar 27;10(3):e1004254. doi: 10.1371/journal.pgen.1004254 (PMC3967955; doi:10.1371/journal.pgen.1004254)

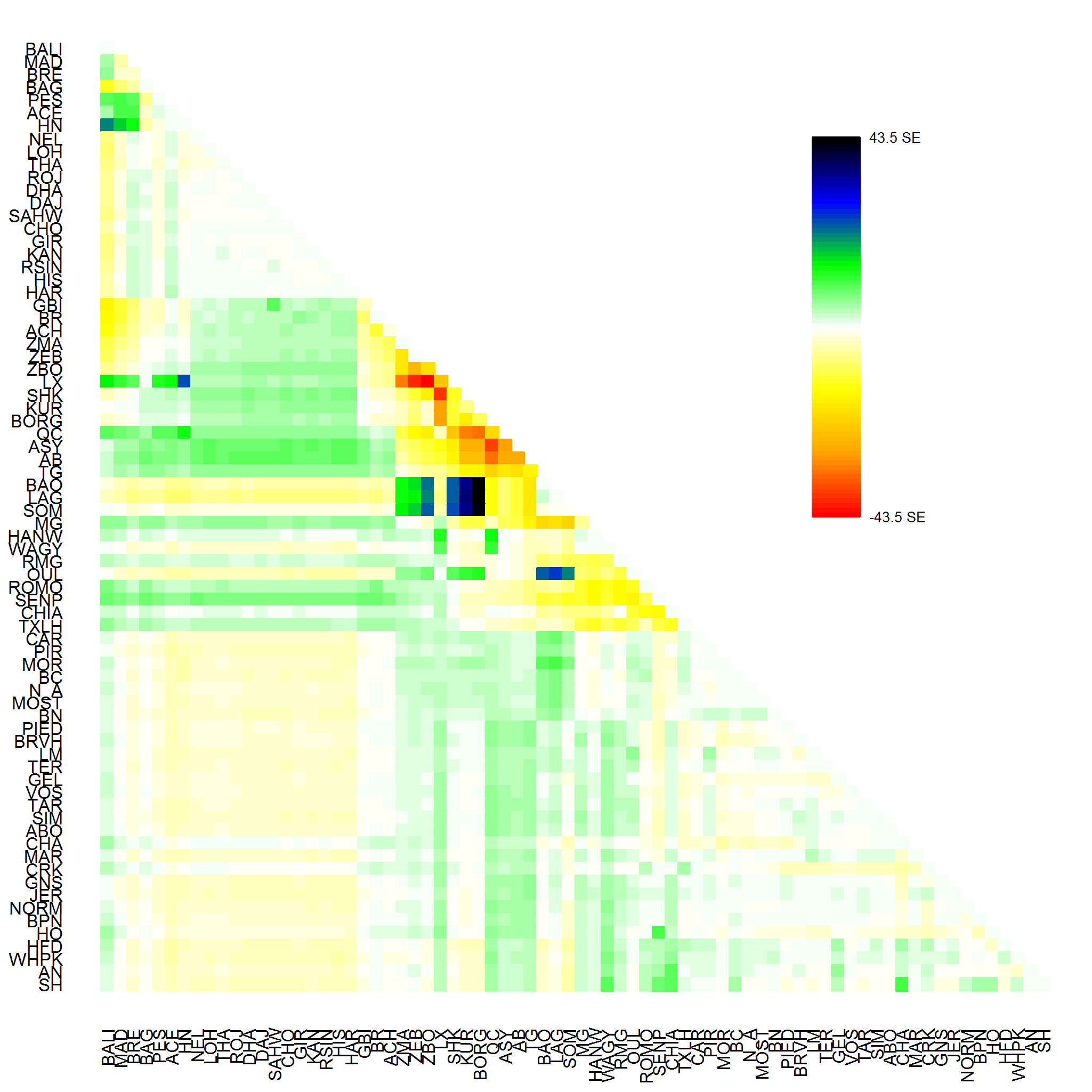

Supplement: Figure S1 — Plot of residuals from the phylogeny model depicted in Figure 3 when no migration edges were fit. (TIF) [file pgen.1004254.s001.tif]

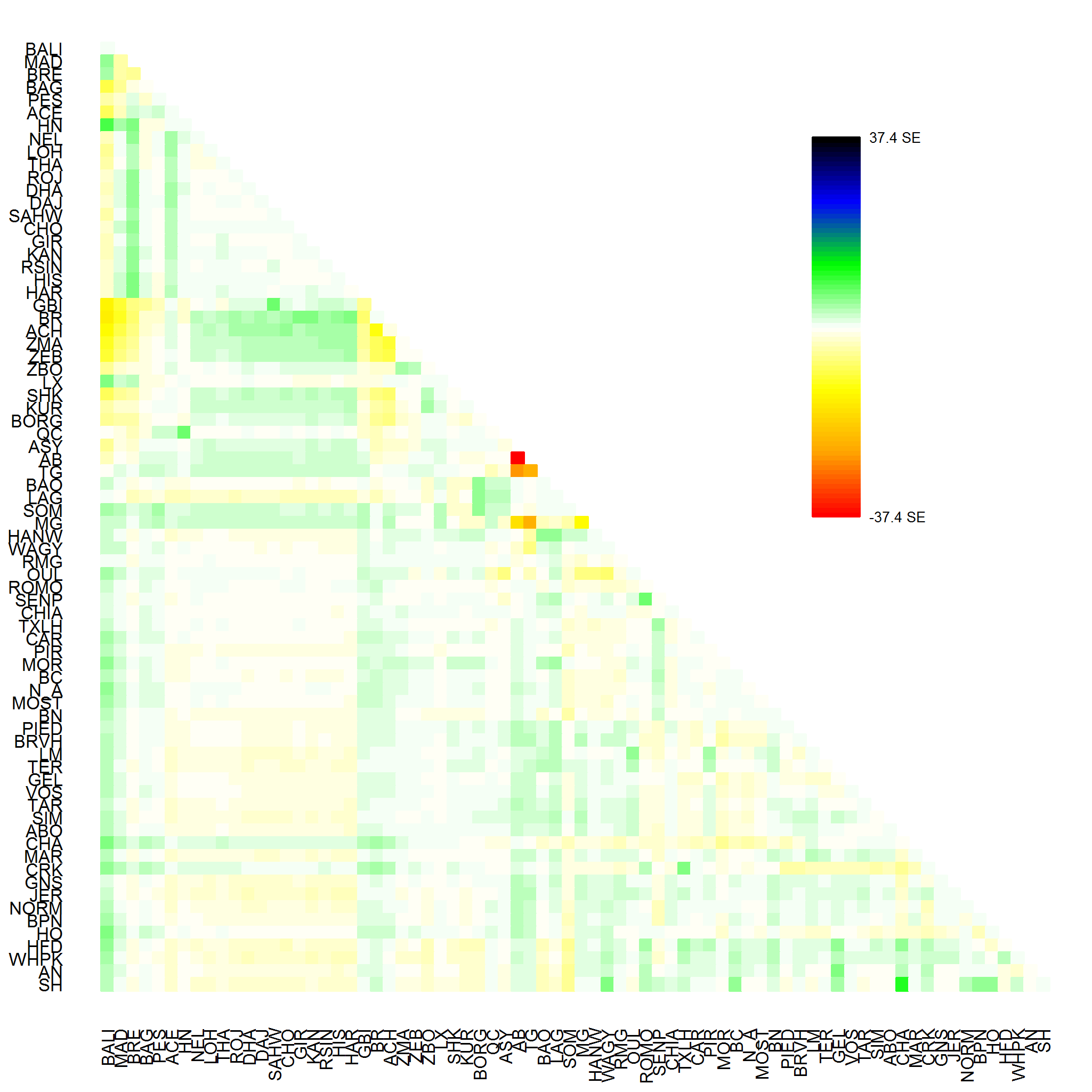

Supplement: Figure S2 — Plot of residuals from the phylogenetic network model depicted in Figure 4 when 17 migration edges were fit. (TIF) [file pgen.1004254.s002.tif]

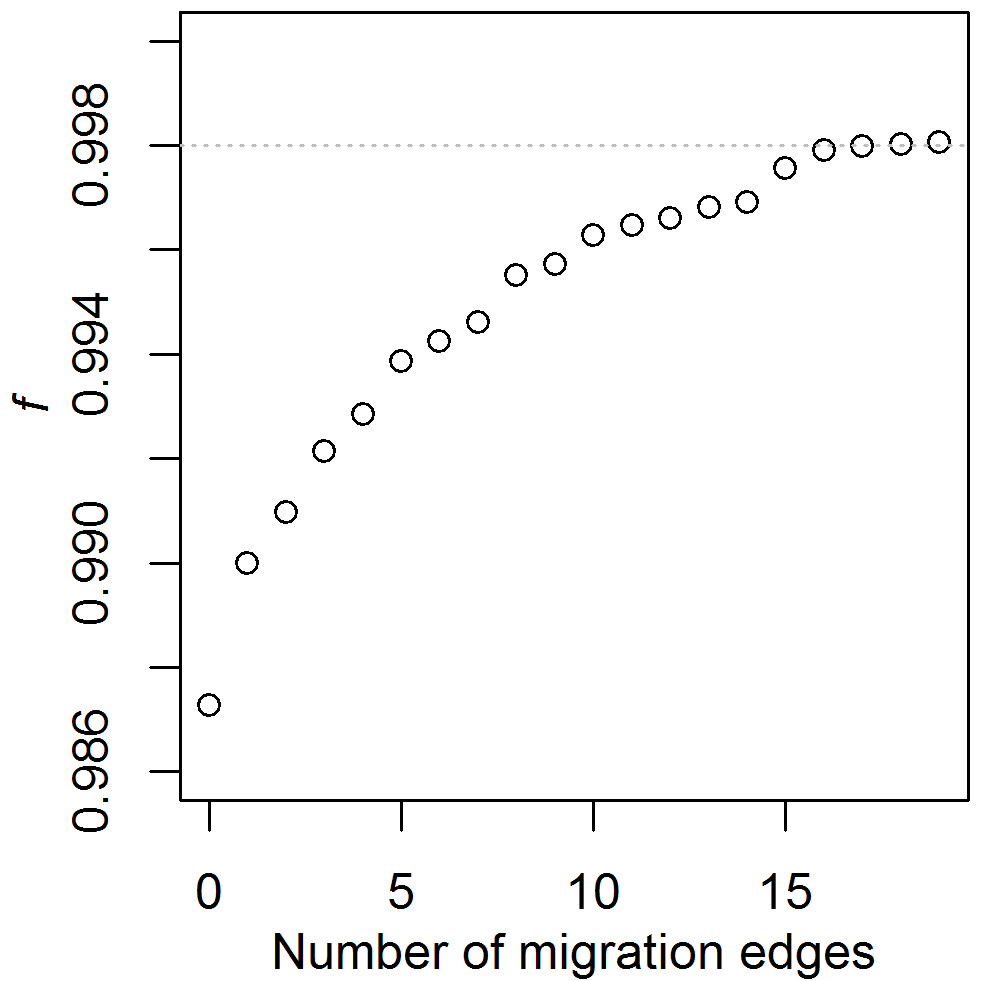

Supplement: Figure S3 — The fraction of variance in relatedness between populations accounted for by phylogenetic models with 0 through 19 migrations. The fraction of variance in the sample covariance matrix () accounted for by the model covariance matrix (). Pickrell and Pritchard [20] showed that the fraction began to asymptote at 0.998 when the models accurately depicted relationships between simulated populations. We also observed this asymptote near 0.998 in our empirical analysis, leading us to conclude that the relationships between the 74 cattle breeds were accurately described by a phylogenetic network with 17 migration edges. (TIF) [file pgen.1004254.s003.tif]

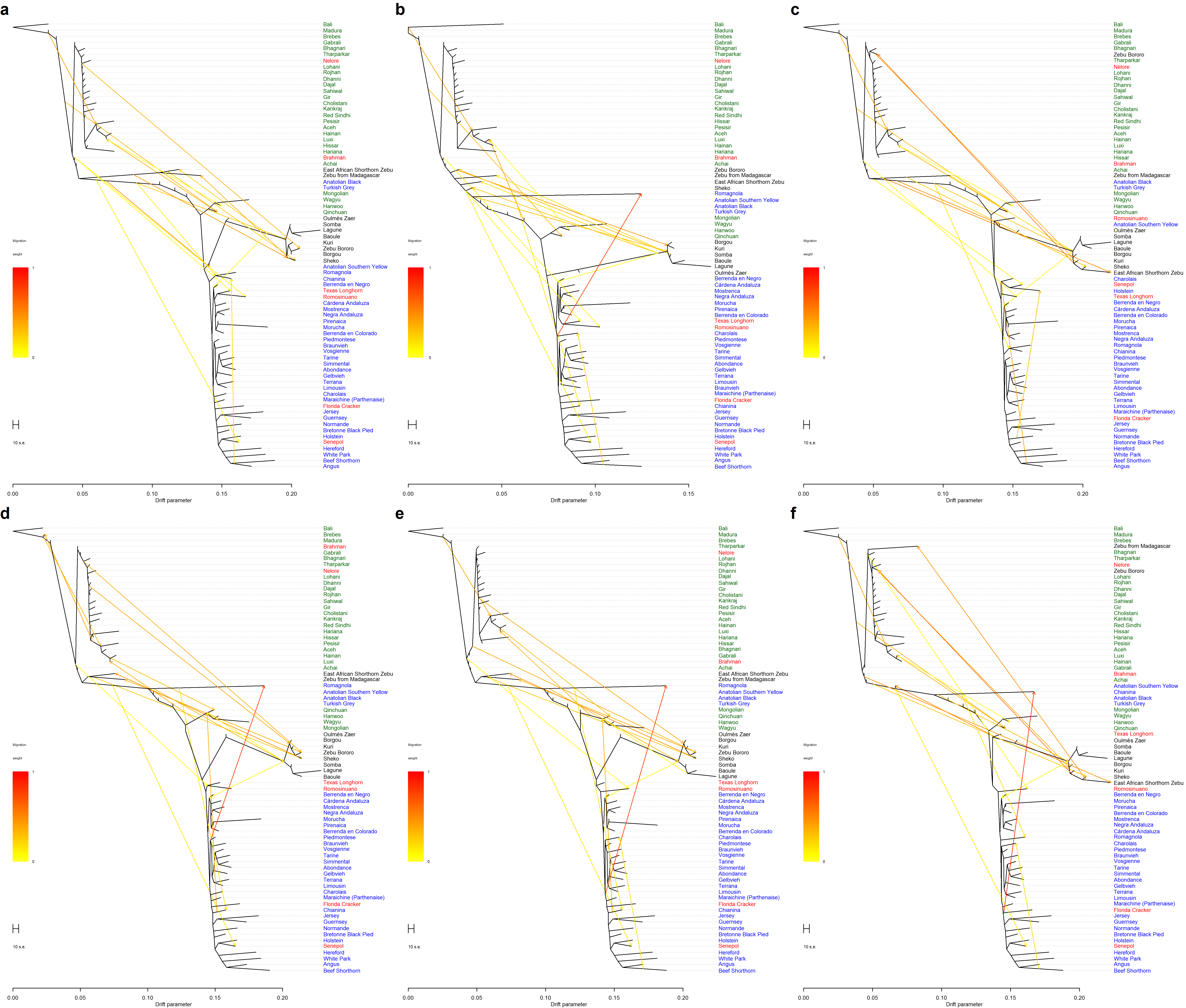

Supplement: Figure S4 — Phylogenetic network with 17 edges (Figure 4) plus 5 independent replicates. Replicates were run with different random seeds to visually evaluate consistency of migration edges. Network a is the same as Figure 4; networks b through f are replicates. Breeds were colored according to their geographic origin; black: Africa, green: Asia, red: North and South America, orange: Australia, and blue: Europe. Scale bar shows 10 times the average standard error of the estimated entries in the sample covariance matrix. Migration edges were colored according to percent ancestry received from the donor population. (TIF) [file pgen.1004254.s004.tif]

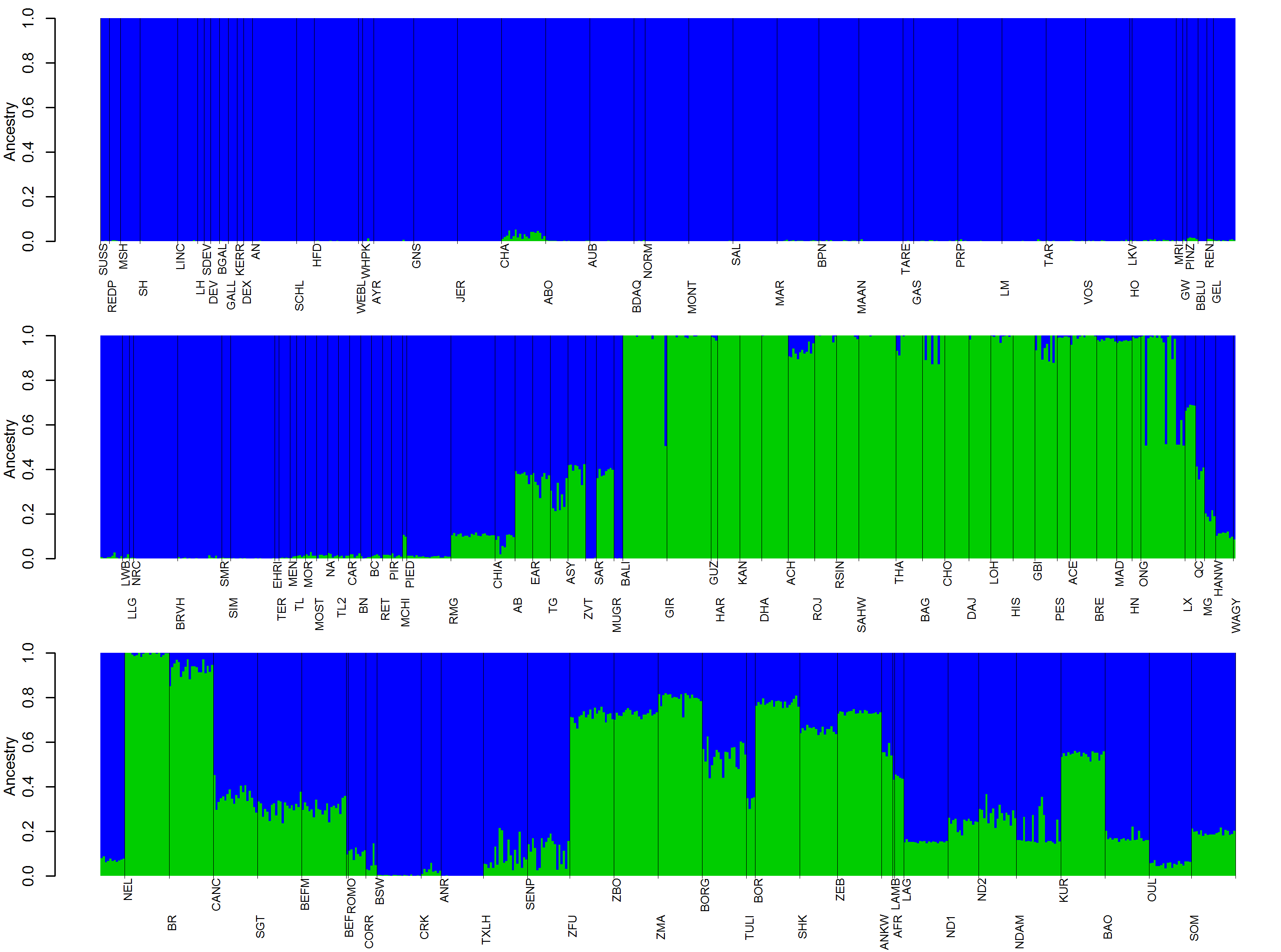

Supplement: Figure S5 — Ancestry models with 2 ancestral populations (K = 2). Blue represents Bos t. taurus ancestry, and green represents Bos javanicus and Bos t. indicus ancestry. (TIF) [file pgen.1004254.s005.tif]

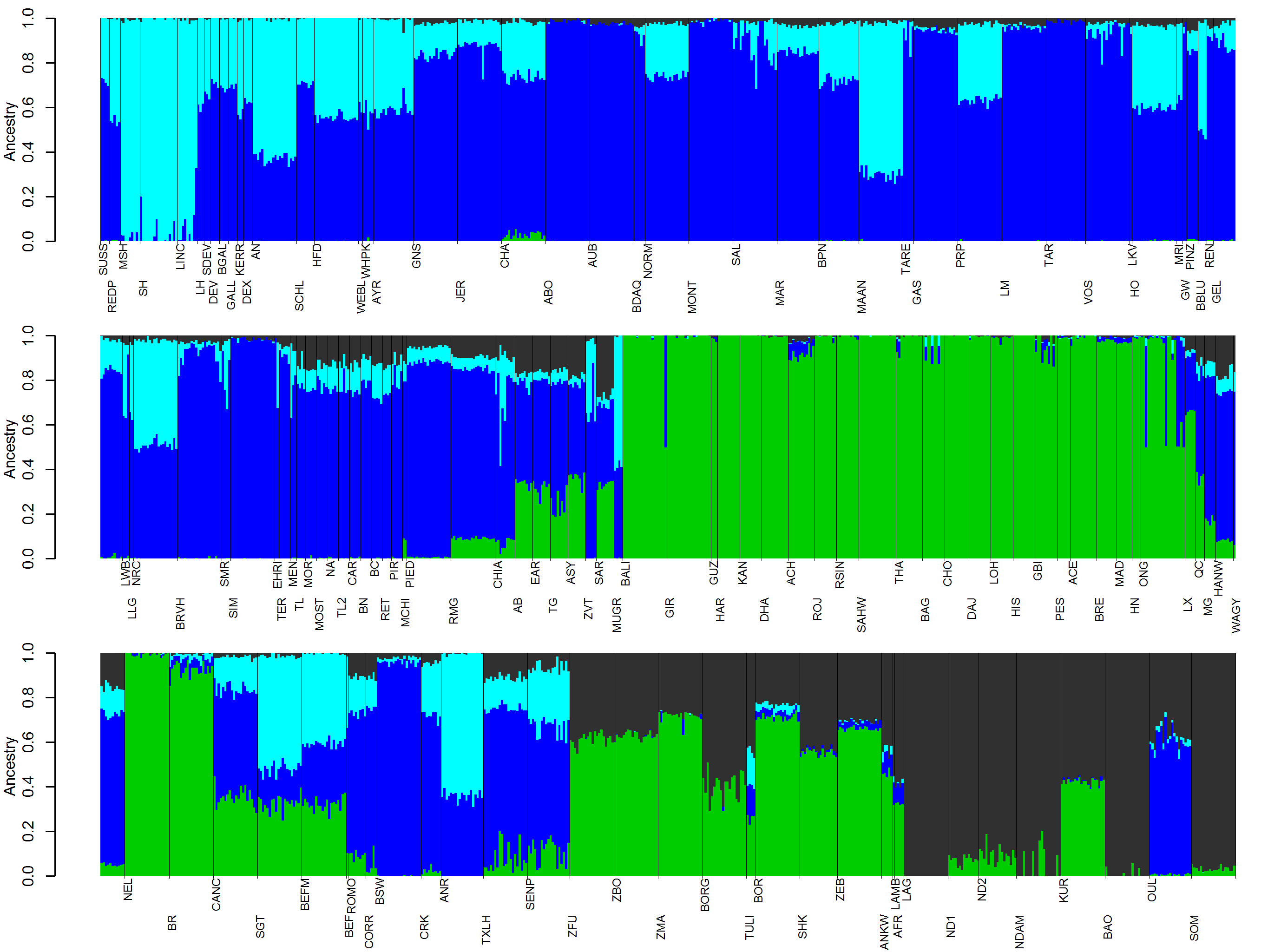

Supplement: Figure S6 — Ancestry models with 4 ancestral populations (K = 4). Blue represents Eurasian Bos t. taurus ancestry, green represents Bos javanicus and Bos t. indicus ancestry, dark grey represents African Bos. t. taurus ancestry, and cyan represents ancestry similar to Durham Shorthorns. (TIF) [file pgen.1004254.s006.tif]

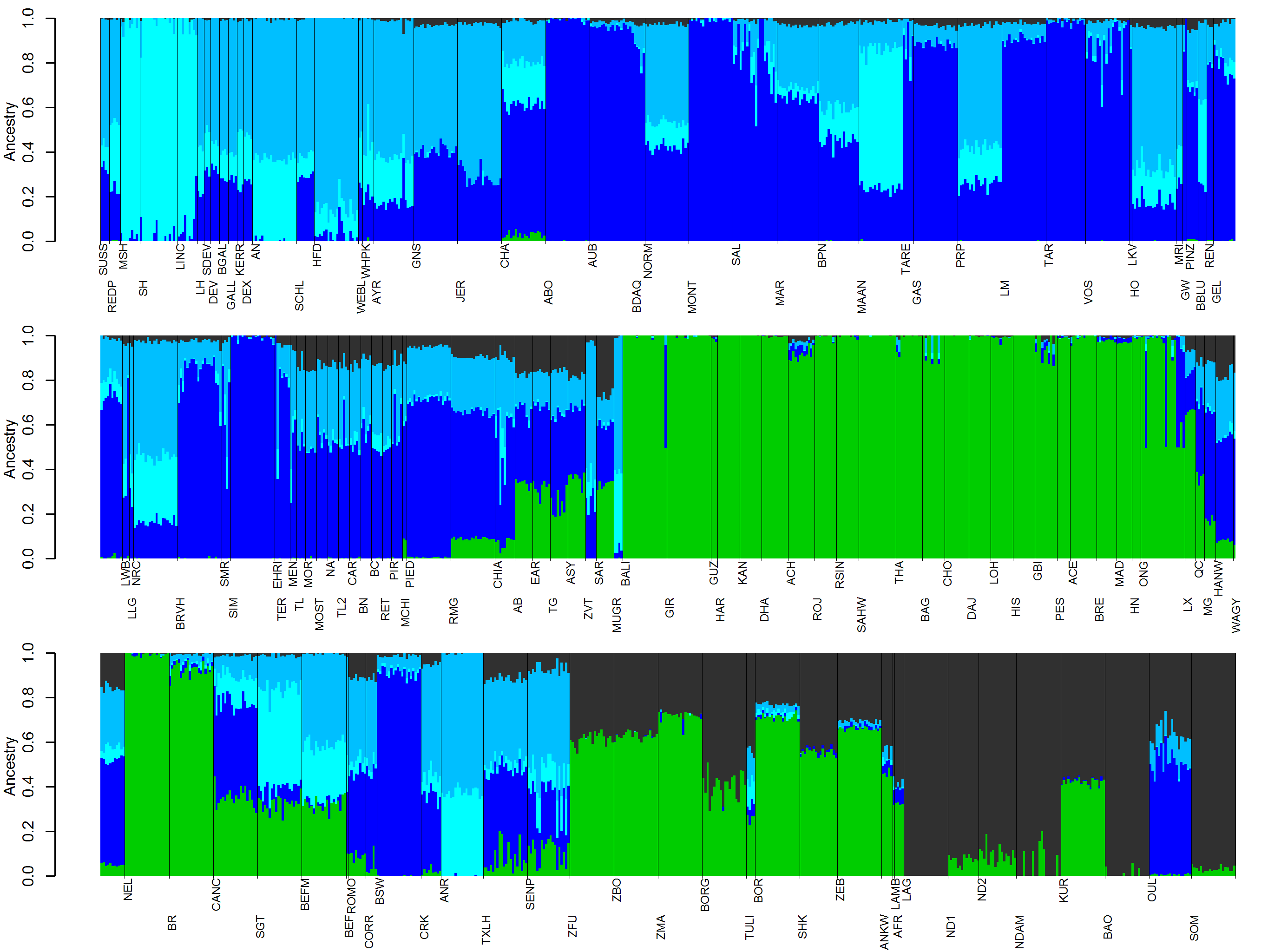

Supplement: Figure S7 — Ancestry models with 5 ancestral populations (K = 5). Blue represents Eurasian Bos t. taurus ancestry, green represents Bos javanicus and Bos t. indicus ancestry, dark grey represents African Bos. t. taurus ancestry, cyan represents ancestry similar to Durham Shorthorns, and deep sky blue represents British and Northern European ancestry. (TIF) [file pgen.1004254.s007.tif]

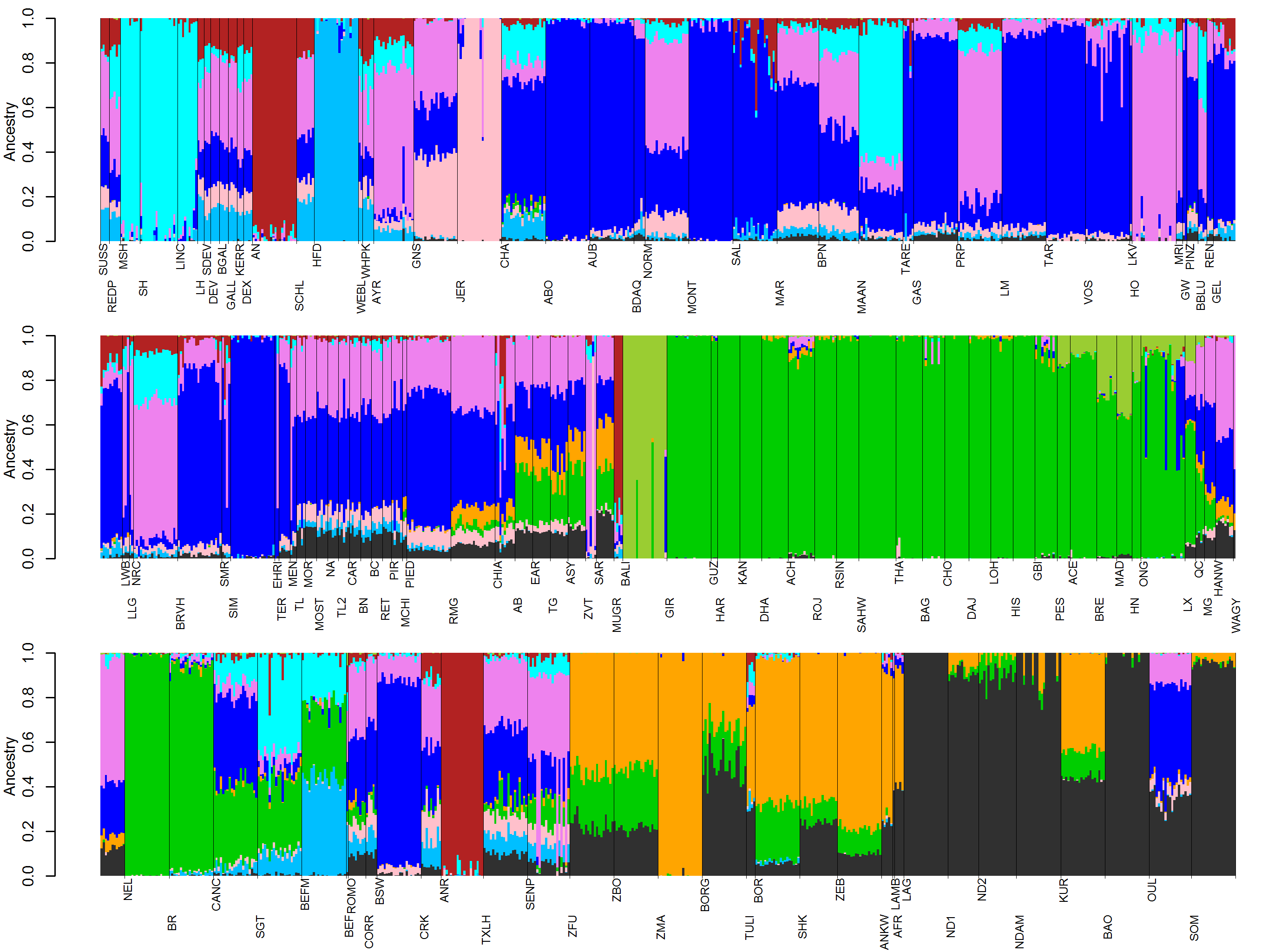

Supplement: Figure S8 — Ancestry models with 10 ancestral populations (K = 10). (TIF) [file pgen.1004254.s008.tif]

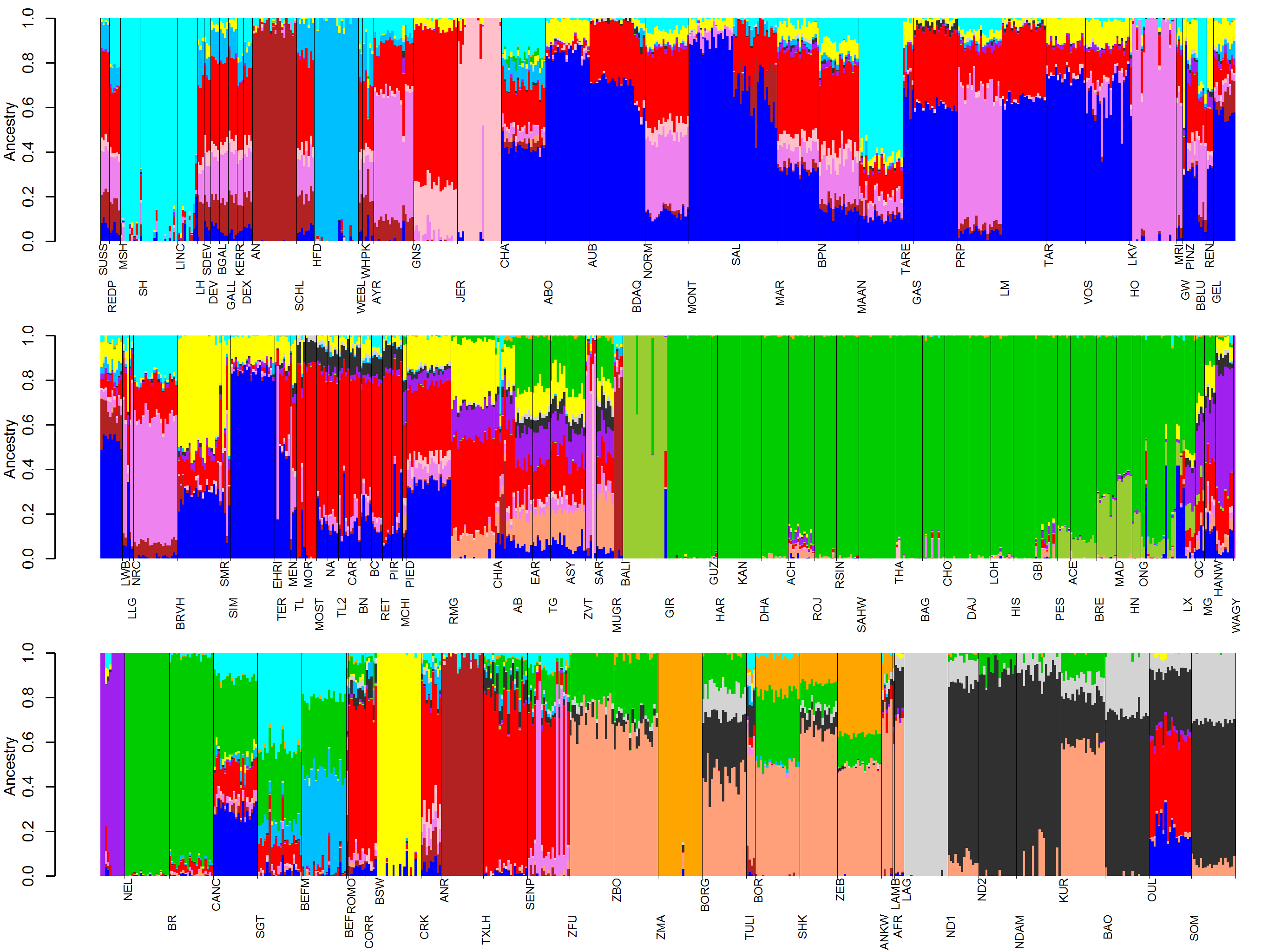

Supplement: Figure S9 — Ancestry models with 15 ancestral populations (K = 15). (TIF) [file pgen.1004254.s009.tif]

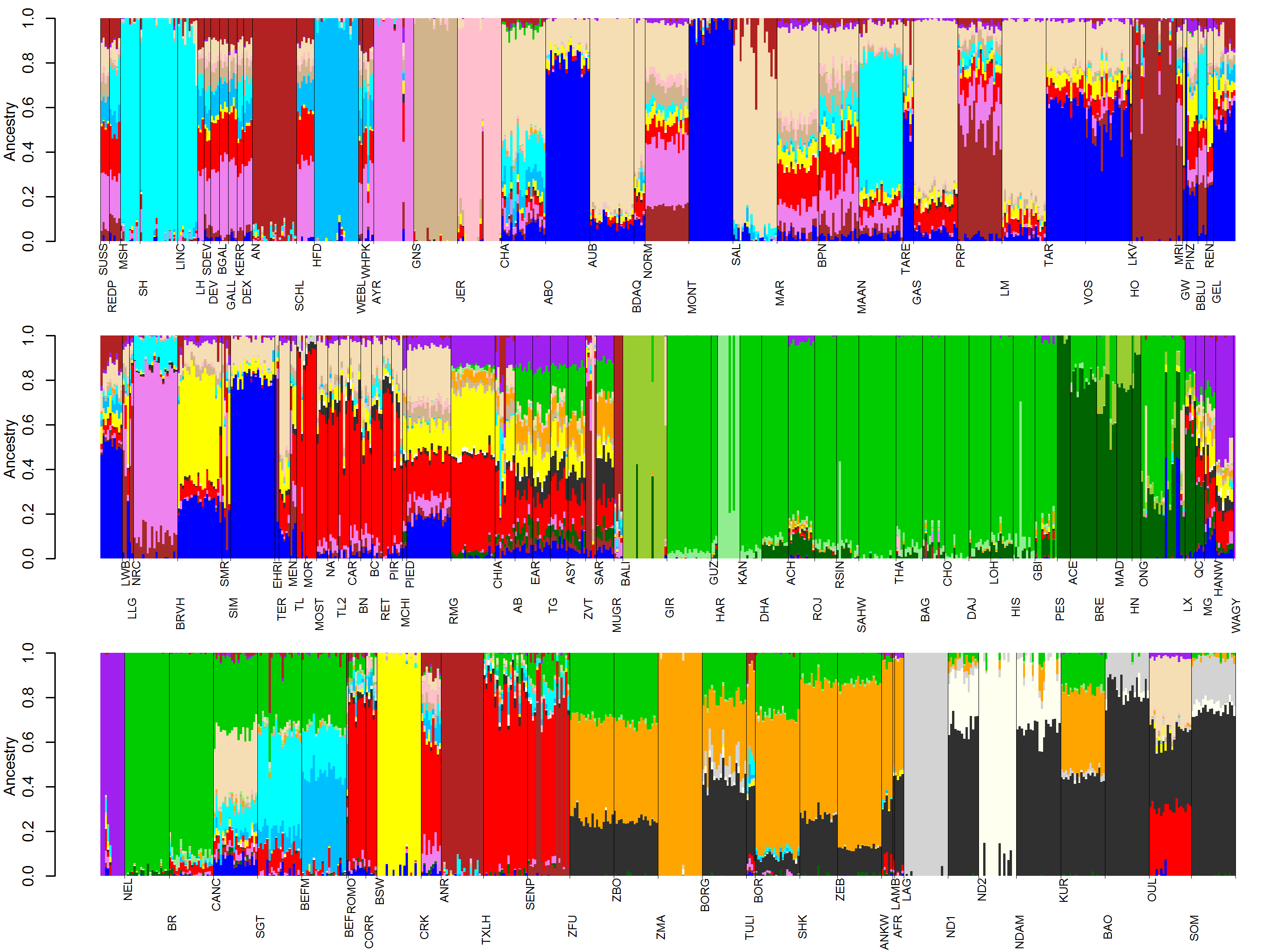

Supplement: Figure S10 — Ancestry models with 20 ancestral populations (K = 20). (TIF) [file pgen.1004254.s010.tif]
