# Supplementary material for: Worldwide Patterns of Ancestry, Divergence, and Admixture in Domesticated Cattle
Source: PLoS Genet. 2014 Mar 27;10(3):e1004254. doi: 10.1371/journal.pgen.1004254 (PMC3967955; doi:10.1371/journal.pgen.1004254)
Supplement: Table S1 — Provenance for all samples included in the analyses. Species and subspecies assignments are according to [25]. (DOC) [file pgen.1004254.s011.doc]

**Table S1. Provenance for all samples included in the analyses.** Species and subspecies assignments are according to Felius (1995).

| **Breed** | **Breed Code** | **No. Samples** | **No. from Decker *et al*. 2009** | **No. from Gautier *et al*. 2010** | **(Sub)Species** | **Continent** | **Geographic Origin** |
| --- | --- | --- | --- | --- | --- | --- | --- |
| Bali | BALI | 20 |  |  | *Bos javanicus* | Asia | Bali, Indonesia |
| Zebu Fulani | ZFU | 20 |  | 20 | *Bos t. indicus* | Africa | Benin |
| Zebu Bororo | ZBO | 20 |  | 20 | *Bos t. indicus* | Africa | Chad |
| Zebu from Madagascar | ZMA | 20 |  | 20 | *Bos t. indicus* | Africa | Madagascar |
| Nelore | NEL | 20 | 5 |  | *Bos t. indicus* | Americas | Brazil |
| Brahman | BR | 20 |  |  | *Bos t. indicus* | Americas | Gulf Coast, United States |
| Ongole Grade | ONG | 20 |  |  | *Bos t. indicus* | Asia | Andhra Pradesh, India |
| Achai | ACH | 12 |  |  | *Bos t. indicus* | Asia | Khyber Pakhtun Khwa, Pakistan |
| Red Sindhi | RSIN | 10 |  |  | *Bos t. indicus* | Asia | Sindh, Pakistan |
| Cholistani | CHO | 11 |  |  | *Bos t. indicus* | Asia | Cholistan Desert, Punjab, Pakistan |
| Gir | GIR | 20 | 9 |  | *Bos t. indicus* | Asia | Gujerat, India |
| Guzerat | GUZ | 3 | 3 |  | *Bos t. indicus* | Asia | Guzarat, India |
| Hariana | HAR | 10 |  |  | *Bos t. indicus* | Asia | Haryana plains, India |
| Brebes | BRE | 9 |  |  | *Bos t. indicus* | Asia | Indonesia |
| Pesisir | PES | 6 |  |  | *Bos t. indicus* | Asia | Indonesia |
| Dajal | DAJ | 10 |  |  | *Bos t. indicus* | Asia | Punjab, Pakistan |
| Bhagnari | BAG | 10 |  |  | *Bos t. indicus* | Asia | Kaochi, Kalat, and Baluchistan, Pakistan |
| Gabrali | GBI | 10 |  |  | *Bos t. indicus* | Asia | Khyber Pakhtun Khwa, Pakistan |
| Hainan | HN | 4 |  |  | *Bos t. indicus* | Asia | Hainan Province, China |
| Madura | MAD | 7 |  |  | *Bos t. indicus* | Asia | Madura Island, Indonesia |
| Kankraj | KAN | 10 |  |  | *Bos t. indicus* | Asia | North Gujerat, India |
| Lohani | LOH | 10 |  |  | *Bos t. indicus* | Asia | Northwest Pakistan |
| Dhanni | DHA | 12 |  |  | *Bos t. indicus* | Asia | Punjab, Pakistan |
| Hissar | HIS | 10 |  |  | *Bos t. indicus* | Asia | Punjab, Pakistan |
| Sahiwal | SAHW | 17 | 10 |  | *Bos t. indicus* | Asia | Punjab, Pakistan |
| Tharparkar | THA | 12 |  |  | *Bos t. indicus* | Asia | Southeast Sindh, Pakistan |
| Rojhan | ROJ | 10 |  |  | *Bos t. indicus* | Asia | Punjab, Pakistan |
| Aceh | ACE | 12 |  |  | *Bos t. indicus* | Asia | Sumatra, Indonesia |
| Lagune | LAG | 20 |  | 20 | *Bos t. taurus* | Africa | Benin |
| Baoule | BAO | 20 |  | 20 | *Bos t. taurus* | Africa | Burkina Faso |
| Kuri | KUR | 20 |  | 20 | *Bos t. taurus* | Africa | Chad |
| N'Dama | NDAM | 20 | 4 |  | *Bos t. taurus* | Africa | Ivory Coast, Africa |
| Oulmès Zaer | OUL | 19 |  | 19 | *Bos t. taurus* | Africa | Morocco |
| N'Dama | ND1 | 14 |  | 14 | *Bos t. taurus* | Africa | Southeast Burkina Faso |
| N'Dama | ND2 | 17 |  | 17 | *Bos t. taurus* | Africa | Southwest Burkina Faso |
| Somba | SOM | 20 |  | 20 | *Bos t. taurus* | Africa | Togo |
| Romosinuano | ROMO | 8 | 8 |  | *Bos t. taurus* | Americas | Columbia |
| Florida Cracker | CRK | 9 |  |  | *Bos t. taurus* | Americas | Florida, United States |
| Corriente | CORR | 5 | 5 |  | *Bos t. taurus* | Americas | Sonora, Mexico |
| Texas Longhorn | TXLH | 20 | 8 |  | *Bos t. taurus* | Americas | Texas, United States |
| Brown Swiss | BSW | 20 | 7 | 8 | *Bos t. taurus* | Americas | United States |
| Red Angus | ANR | 19 | 6 |  | *Bos t. taurus* | Americas | United States |
| Senepol | SENP | 19 |  |  | *Bos t. taurus* | Americas | United States Virgin Islands |
| Wagyu | WAGY | 12 | 6 |  | *Bos t. taurus* | Asia | Japan |
| Hanwoo | HANW | 8 |  |  | *Bos t. taurus* | Asia | Korea |
| Mongolian | MG | 5 |  |  | *Bos t. taurus* | Asia | Mongolia1 |
| Murray Grey | MUGR | 4 | 4 |  | *Bos t. taurus* | Australia | Australia |
| Angus | AN | 20 | 1 |  | *Bos t. taurus* | Europe | Aberdeenshire, Scotland |
| Tarentaise | TARE | 5 | 5 |  | *Bos t. taurus* | Europe | Alpine Massif-Central of southeastern France |
| Pinzgauer | PINZ | 5 | 5 |  | *Bos t. taurus* | Europe | Austria |
| Belgian Blue | BBLU | 4 | 4 |  | *Bos t. taurus* | Europe | Belgium |
| Simmental | SIM | 20 |  |  | *Bos t. taurus* | Europe | Bern, Switzerland |
| Simmentaler | SMR | 4 |  |  | *Bos t. taurus* | Europe | Bern, Switzerland |
| Maine-Anjou | MAAN | 20 | 5 | 15 | *Bos t. taurus* | Europe | Brittany, France |
| Rendena | REN | 3 |  |  | *Bos t. taurus* | Europe | Central Alps |
| Gelbvieh | GEL | 20 | 5 |  | *Bos t. taurus* | Europe | Central Germany |
| Berrenda en Negro | BN | 5 |  |  | *Bos t. taurus* | Europe | Ciudad Real, Jaen, Cordoba, Sevilla, and Huelva, Spain |
| Berrenda en Colorado | BC | 5 |  |  | *Bos t. taurus* | Europe | Cordoba, Sevilla, Huelva, and Cadiz, Spain |
| Devon | DEV | 4 | 4 |  | *Bos t. taurus* | Europe | Devon, England |
| Romagnola | RMG | 20 | 10 |  | *Bos t. taurus* | Europe | Emilia, Italy |
| Beef Shorthorn | SH | 17 | 7 |  | *Bos t. taurus* | Europe | England |
| Lincoln Red | LINC | 9 | 9 |  | *Bos t. taurus* | Europe | England |
| Milking Shorthorn | MSH | 9 | 1 |  | *Bos t. taurus* | Europe | England |
| Montbeliard | MONT | 20 | 2 | 18 | *Bos t. taurus* | Europe | France |
| Normande | NORM | 20 | 1 | 19 | *Bos t. taurus* | Europe | France |
| Guernsey | GNS | 20 | 10 |  | *Bos t. taurus* | Europe | Guernsey Island |
| Holstein | HO | 20 |  |  | *Bos t. taurus* | Europe | Holland |
| Dexter | DEX | 4 | 4 |  | *Bos t. taurus* | Europe | Ireland |
| Kerry | KERR | 3 | 3 |  | *Bos t. taurus* | Europe | Ireland |
| Marchigiana | MCHI | 2 | 2 |  | *Bos t. taurus* | Europe | Italy |
| Jersey | JER | 20 | 7 |  | *Bos t. taurus* | Europe | Jersey Island |
| Lithuanian Light Grey | LLG | 2 |  |  | *Bos t. taurus* | Europe | Lithuania |
| Lithuanian White Backed | LWB | 3 |  |  | *Bos t. taurus* | Europe | Lithuania |
| Limousin | LM | 20 |  |  | *Bos t. taurus* | Europe | Massif Central, France |
| Salers | SAL | 20 | 4 |  | *Bos t. taurus* | Europe | Massif Central, France |
| Menorquina | MEN | 3 |  |  | *Bos t. taurus* | Europe | Menorca, Spain |
| Mostrenca | MOST | 5 |  |  | *Bos t. taurus* | Europe | National Park of Donana, southwestern Spain |
| Groningen Whitehead | GW | 2 |  |  | *Bos t. taurus* | Europe | Netherlands |
| Lakenvelder | LKV | 1 |  |  | *Bos t. taurus* | Europe | Netherlands |
| Meuse-Rhine-Ijjsel | MRI | 3 |  |  | *Bos t. taurus* | Europe | Netherlands |
| Red Poll | REDP | 5 | 5 |  | *Bos t. taurus* | Europe | Norfolk and Suffolk, England |
| Vosgienne | VOS | 20 |  | 20 | *Bos t. taurus* | Europe | Northeast France |
| Bretonne Black Pied | BPN | 18 |  | 18 | *Bos t. taurus* | Europe | Northwest France |
| French Red Pied Lowland | PRP | 20 |  | 20 | *Bos t. taurus* | Europe | Northwest France |
| Maraichine (Parthenaise) | MAR | 19 |  | 19 | *Bos t. taurus* | Europe | Northwest France |
| Piedmontese | PIED | 20 | 9 |  | *Bos t. taurus* | Europe | Northwest Italy |
| Pirenaica | PIR | 5 |  |  | *Bos t. taurus* | Europe | northwest of Spain |
| Norwegian Red | NRC | 20 | 9 |  | *Bos t. taurus* | Europe | Norway |
| Blonde d'Aquitaine | BDAQ | 5 | 5 |  | *Bos t. taurus* | Europe | Pyrenees, France |
| Morucha | MOR | 5 |  |  | *Bos t. taurus* | Europe | Salamanca |
| Charolais | CHA | 20 |  |  | *Bos t. taurus* | Europe | Saône-et-Loire, France |
| Belted Galloway | BGAL | 4 | 4 |  | *Bos t. taurus* | Europe | Scotland |
| Galloway | GALL | 4 | 4 |  | *Bos t. taurus* | Europe | Scotland |
| Finnish Ayrshire | AYR | 18 | 2 |  | *Bos t. taurus* | Europe | Scotland/Finland |
| Negra Andaluza | NA | 5 |  |  | *Bos t. taurus* | Europe | Sierra Morena Mountains, Cordoba, and Sevilla Spain |
| Cardena Andaluza | CAR | 5 |  |  | *Bos t. taurus* | Europe | Sierra Morena, Spain |
| South Devon | SDEV | 3 | 3 |  | *Bos t. taurus* | Europe | South England |
| Aubrac | AUB | 20 |  | 20 | *Bos t. taurus* | Europe | South France |
| Sussex | SUSS | 4 | 4 |  | *Bos t. taurus* | Europe | Southeast England |
| Abondance | ABO | 20 |  | 20 | *Bos t. taurus* | Europe | Southeast France |
| Tarine | TAR | 18 |  | 18 | *Bos t. taurus* | Europe | Southeast France |
| Gascon | GAS | 20 |  | 20 | *Bos t. taurus* | Europe | Southwest France |
| Retinta | RET | 4 |  |  | *Bos t. taurus* | Europe | Southwest of Spain and bordering Portugal |
| Toro de Lidia | TL | 4 |  |  | *Bos t. taurus* | Europe | Spain |
| Toro de Lidia | TL2 | 5 |  |  | *Bos t. taurus* | Europe | Spain |
| Braunvieh | BRVH | 20 |  |  | *Bos t. taurus* | Europe | Switzerland |
| Ehringer | EHRI | 2 |  |  | *Bos t. taurus* | Europe | Switzerland |
| Anatolian Black | AB | 8 |  |  | *Bos t. taurus* | Europe | Turkey |
| Anatolian Southern Yellow | ASY | 8 |  |  | *Bos t. taurus* | Europe | Turkey |
| East Anatolian Red | EAR | 8 |  |  | *Bos t. taurus* | Europe | Turkey |
| South Anatolian Red | SAR | 8 |  |  | *Bos t. taurus* | Europe | Turkey |
| Turkish Grey | TG | 8 |  |  | *Bos t. taurus* | Europe | Turkey |
| Zavot | ZVT | 5 |  |  | *Bos t. taurus* | Europe | Turkey |
| Terrana | TER | 5 |  |  | *Bos t. taurus* | Europe | Vasconcades mountainous region of Alava, Spain |
| Hereford | HFD | 20 | 1 |  | *Bos t. taurus* | Europe | Wales |
| Welsh Black | WEBL | 2 | 2 |  | *Bos t. taurus* | Europe | Wales |
| White Park | WHPK | 5 | 4 |  | *Bos t. taurus* | Europe | Wales |
| Chianina | CHIA | 9 | 7 |  | *Bos t. taurus* | Europe | West Central Italy |
| Scottish Highland | SCHL | 8 | 8 |  | *Bos t. taurus* | Europe | Western Scottland |
| Longhorn | LH | 3 | 3 |  | *Bos t. taurus* | Europe | Yorkshire, England |
| Borgou | BORG | 20 |  | 20 | Hybrid | Africa | Benin |
| Tuli | TULI | 4 |  |  | Hybrid | Africa | Botswana |
| Landim | LAMB | 1 |  |  | Hybrid | Africa | East Coast of South Africa |
| Sheko | SHK | 17 |  |  | Hybrid | Africa | Ethiopia |
| East African Shorthorn Zebu | ZEB | 20 |  |  | Hybrid | Africa | Kenya |
| Ankole-Watusi | ANKW | 5 |  |  | Hybrid | Africa | Ruanda |
| Africander | AFR | 4 |  |  | Hybrid | Africa | South Africa |
| Boran | BOR | 20 |  |  | Hybrid | Africa | southern Ethiopia |
| Canchim | CANC | 20 |  |  | Hybrid | Americas | Brazil |
| Beefalo | BEF | 1 |  |  | Hybrid | Americas | Northwest United States |
| Beefmaster | BEFM | 20 |  |  | Hybrid | Americas | Texas, United States |
| Santa Gertrudis | SGT | 20 |  |  | Hybrid | Americas | Texas, United States |
| Luxi | LX | 5 |  |  | Hybrid | Asia | Shandong Province, China |
| Qinchuan | QC | 4 |  |  | Hybrid | Asia | Shaanxi Province, China |
