# Supplementary material for: Worldwide Patterns of Ancestry, Divergence, and Admixture in Domesticated Cattle
Source: PLoS Genet. 2014 Mar 27;10(3):e1004254. doi: 10.1371/journal.pgen.1004254 (PMC3967955; doi:10.1371/journal.pgen.1004254)
Supplement: Table S2 — Cross-validation and ΔK values for ADMIXTURE ancestry models with K ranging from 1 to 20. (DOC) [file pgen.1004254.s012.doc]

**Table S2.** **Cross-validation and *ΔK* values for ADMIXTURE ancestry models with *K* ranging from 1 to 20.**

| ***K*** | **Cross-validation error** | **Log-likelihood** | ***L*'(*K*)** | ***L*''(*K*)** |
| --- | --- | --- | --- | --- |
| 1 | 0.63636 | -65702704 | N/A | N/A |
| 2 | 0.54374 | -59538328 | 6164375.96 | 4545316.79 |
| 3 | 0.51985 | -57919269 | 1619059.17 | 1105462.91 |
| 4 | 0.51288 | -57405673 | 513596.27 | 253401.84 |
| 5 | 0.50983 | -57145478 | 260194.42 | 26732.82 |
| 6 | 0.50693 | -56912017 | 233461.61 | 9346.55 |
| 7 | 0.50424 | -56687902 | 224115.05 | -163722.93 |
| 8 | 0.49888 | -56300064 | 387837.99 | 450205.28 |
| 9 | 0.50094 | -56362431 | -62367.29 | -547943.87 |
| 10 | 0.49428 | -55876855 | 485576.58 | 382526.32 |
| 11 | 0.49325 | -55773804 | 103050.26 | -68045.84 |
| 12 | 0.49175 | -55602708 | 171096.10 | 46476.86 |
| 13 | 0.49041 | -55478089 | 124619.24 | -79680.06 |
| 14 | 0.48836 | -55273790 | 204299.30 | 81316.25 |
| 15 | 0.48708 | -55150807 | 122983.05 | 37711.26 |
| 16 | 0.48640 | -55065535 | 85271.79 | -65609.06 |
| 17 | 0.48497 | -54914654 | 150880.84 | 72573.21 |
| 18 | 0.48455 | -54836346 | 78307.63 | -63149.16 |
| 19 | 0.48293 | -54694890 | 141456.79 | 38935.55 |
| 20 | 0.48206 | -54592368 | 102521.24 | 102521.24 |
