# Supplementary material for: Worldwide Patterns of Ancestry, Divergence, and Admixture in Domesticated Cattle
Source: PLoS Genet. 2014 Mar 27;10(3):e1004254. doi: 10.1371/journal.pgen.1004254 (PMC3967955; doi:10.1371/journal.pgen.1004254)
Supplement: Table S3 — Five most negative and significant f3 statistics for Brebes and Madura showing Bali (Bos javanicus) introgression. (DOC) [file pgen.1004254.s013.doc]

**Table S3. Five most negative and significant *f_3_* statistics for Brebes and Madura showing Bali (*Bos javanicus***) introgression.

| **Population A** | **Population B** | **Population C** | ***f_3_*** | **Standard Error** | **Z-score** |
| --- | --- | --- | --- | --- | --- |
| Brebes | Bali | Lohani | -0.00765 | 0.00041 | -18.80 |
| Brebes | Nelore | Bali | -0.00769 | 0.00041 | -18.76 |
| Brebes | Red Sindhi | Bali | -0.00747 | 0.00040 | -18.73 |
| Brebes | Brahman | Bali | -0.00708 | 0.00039 | -18.24 |
| Brebes | Sahiwal | Bali | -0.00764 | 0.00042 | -18.07 |
|  |  |  |  |  |  |
| Madura | Bali | Lohani | -0.00656 | 0.00047 | -13.96 |
| Madura | Aceh | Bali | -0.00661 | 0.00048 | -13.86 |
| Madura | Rojhan | Bali | -0.00605 | 0.00046 | -13.09 |
| Madura | Achai | Bali | -0.00601 | 0.00046 | -13.08 |
| Madura | Sahiwal | Bali | -0.00639 | 0.00049 | -13.07 |
