# Supplementary material for: Worldwide Patterns of Ancestry, Divergence, and Admixture in Domesticated Cattle
Source: PLoS Genet. 2014 Mar 27;10(3):e1004254. doi: 10.1371/journal.pgen.1004254 (PMC3967955; doi:10.1371/journal.pgen.1004254)
Supplement: Table S4 — Five most negative and significant f3 statistics for Maine-Anjou, Santa Gertrudis, and Beefmaster showing Shorthorn admixture. (DOC) [file pgen.1004254.s014.doc]

**Table S4. Five most negative and significant *f_3_* statistics for Maine-Anjou, Santa Gertrudis, and Beefmaster showing Shorthorn admixture.**

| **Population A** | **Population B** | **Population C** | ***f_3_*** | **Standard Error** | **Z-score** |
| --- | --- | --- | --- | --- | --- |
| Maine Anjou | Tarine | Beef Shorthorn | -0.00389 | 0.00057 | -6.84 |
| Maine Anjou | Aubrac | Beef Shorthorn | -0.00359 | 0.00056 | -6.47 |
| Maine Anjou | Tarine | Milking Shorthorn | -0.00391 | 0.00061 | -6.36 |
| Maine Anjou | Africander | Beef Shorthorn | -0.00357 | 0.00057 | -6.30 |
| Maine Anjou | Somba | Beef Shorthorn | -0.00376 | 0.00060 | -6.29 |
|  |  |  |  |  |  |
| Santa Gertrudis | Tharparkar | Beef Shorthorn | -0.02641 | 0.00071 | -37.12 |
| Santa Gertrudis | Rojhan | Beef Shorthorn | -0.02664 | 0.00072 | -37.11 |
| Santa Gertrudis | Aceh | Beef Shorthorn | -0.02618 | 0.00071 | -36.80 |
| Santa Gertrudis | Dajal | Beef Shorthorn | -0.02674 | 0.00073 | -36.50 |
| Santa Gertrudis | Kankraj | Beef Shorthorn | -0.02681 | 0.00074 | -36.47 |
|  |  |  |  |  |  |
| Beefmaster | Gir | Beef Shorthorn | -0.02004 | 0.00099 | -20.22 |
| Beefmaster | Beef Shorthorn | Guzerat | -0.01943 | 0.00099 | -19.60 |
| Beefmaster | Dajal | Beef Shorthorn | -0.01965 | 0.00101 | -19.54 |
| Beefmaster | Beef Shorthorn | Dhanni | -0.01965 | 0.00101 | -19.52 |
| Beefmaster | Tharparkar | Beef Shorthorn | -0.01933 | 0.00100 | -19.26 |
